# Management of Malignant Psoas Syndrome From Breast Cancer: A Case Report

**DOI:** 10.7759/cureus.84403

**Published:** 2025-05-19

**Authors:** Maika Yoshioka, Jun Yamamura, Yukiko Miyamura, Yumiko Yasuhara, Shunji Kamigaki

**Affiliations:** 1 Breast and Endocrine Surgery, Sakai City Medical Center, Sakai, JPN; 2 Pathology, Sakai City Medical Center, Sakai, JPN

**Keywords:** invasive pleomorphic lobular carcinoma, luminal like-breast cancer, malignant psoas syndrome, psoas metastasis, skeletal muscle metastasis

## Abstract

Malignant psoas syndrome (MPS) is a cancer-related pain syndrome caused by the metastasis or direct invasion of malignant tumors into the psoas muscle. Breast cancer metastases to skeletal muscles, particularly the psoas muscle, are extremely rare, leading to a limited awareness of MPS. Even suspected MPS is rarely reported in the literature. Here, we present the first confirmed case of psoas muscle metastasis in a patient undergoing treatment for recurrent breast cancer who was diagnosed with MPS using computed tomography-guided biopsy. Early recognition of psoas muscle metastasis is essential for improving patient outcomes, particularly in maintaining daily functional activities.

## Introduction

Malignant psoas syndrome (MPS), first described by Stevens et al. in 1990, is a cancer-related syndrome caused by metastasis or direct invasion of malignant tumors into the psoas muscle [1]. Despite skeletal muscle accounting for about 40% of body weight and being richly vascularized, metastases to muscle are rare. This is thought to be due to physiological barriers such as high tissue pressure, constant mechanical activity, and a local environment that is unfavorable for tumor survival [2].

Breast cancer frequently metastasizes to bone, lung, liver, and brain, but skeletal muscle involvement is extremely rare. MPS is reported in less than 1% of patients with high-risk cancers, and diagnosis can be challenging due to nonspecific symptoms. Moreover, treatment is not standardized, and prognosis is generally poor [3].

Here, we present what appears to be the first pathologically confirmed case of MPS secondary to breast cancer. This case highlights an unusual metastatic pattern and serves as a reminder to consider MPS in patients presenting with persistent muscle pain. We also review previously reported cases to summarize clinical features and diagnostic considerations.

## Case presentation

**Figure 1:**
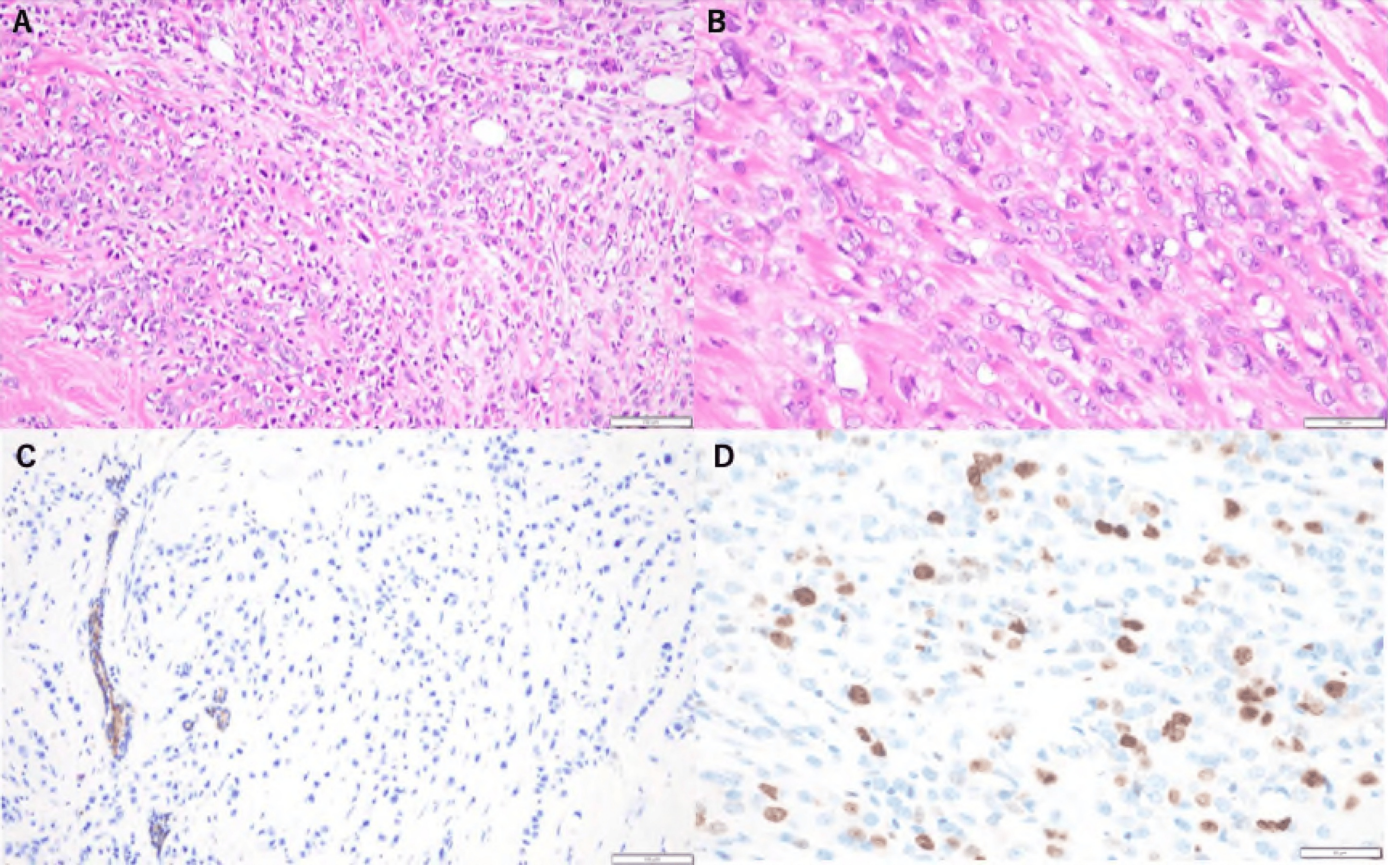
Microscopic morphology of invasive pleomorphic lobular carcinoma (IPLC). (A) Hematoxylin and eosin (H&E) stain (×200). Note the diffuse infiltration of cancer cells. (B) H&E stain (×400). IPLC cells have enlarged nuclei and abundant eosinophilic cytoplasm. (C) E-cadherin stain (×200). Lobular cancer cells are typically negative for E-cadherin stain, and in this patient, the cells are negative. (D) The MIB-1 labeling index, a marker of proliferative activity, was 28% (×400).

A 78-year-old woman with recurrent breast cancer presented with mild throbbing left hip pain. She had been diagnosed with left-sided primary breast cancer eight years previously and had undergone a total mastectomy at that time. Pathological examination confirmed an invasive pleomorphic lobular carcinoma (IPLC), histological grade 2, and an MIB-1 labeling index (a marker of proliferative activity) of 28% (Figure 1).

**Figure 2:**
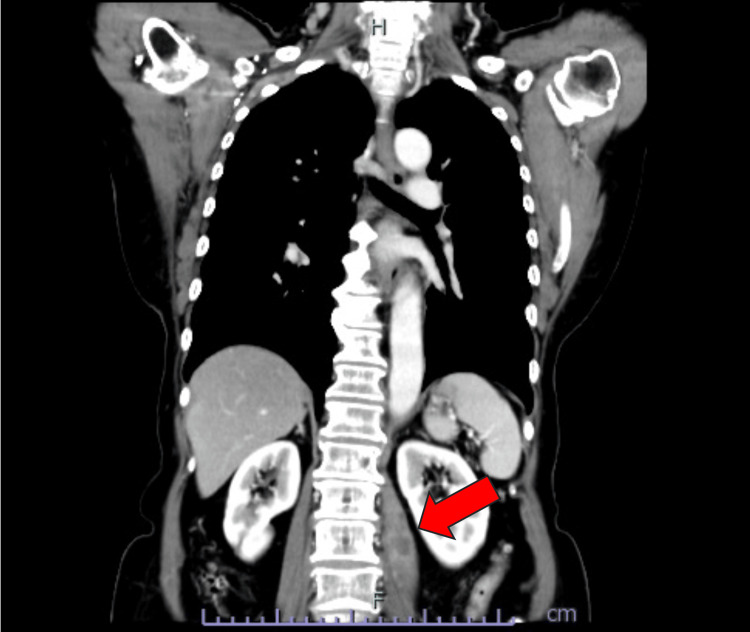
Coronal computed tomography findings. Low-density mass in the left psoas muscle with enhancing peripheral rim (arrow), measuring 65 × 18 mm, thought to likely represent a left psoas abscess.

Immunohistochemical (IHC) analysis revealed the tumor to be estrogen receptor (ER) positive, progesterone receptor (PR) positive, and human epidermal growth factor receptor 2 (HER2) negative. The pathological stage was classified as pT3N1M0/stage IIIA, according to the Union for International Cancer Control (UICC) 8th edition, which incorporates tumor size, nodal status, and distant metastasis. Postoperatively, the patient underwent chemotherapy with fluorouracil 500 mg/m^2^, epirubicin 100 mg/m^2^, cyclophosphamide 500 mg/m^2^, and paclitaxel 80 mg/m^2^, followed by the nonsteroidal aromatase inhibitor anastrozole for five years. Fifteen months after the completion of the initial treatments, a local recurrence was surgically excised from the skin of the left chest wall. Histopathologic examination revealed metastatic carcinoma with ER-positive, PR-positive, and HER2-negative immunostaining similar to the previous breast cancer. The patient was treated with another nonsteroidal aromatase inhibitor, letrozole. Five months later, multiple bone metastases were detected, and tamoxifen and bisphosphonate therapies were initiated. One year after the detection of bone metastases, she began experiencing mild throbbing pain in the left hip, which progressively worsened over several weeks, particularly with walking or bending. On examination of the left hip, there were no obvious abnormalities on inspection or palpation. Motor examination was normal. Contrast-enhanced computed tomography (CT) of the chest and abdomen revealed a low-density mass in the left psoas (Figure 2).

**Figure 3:**
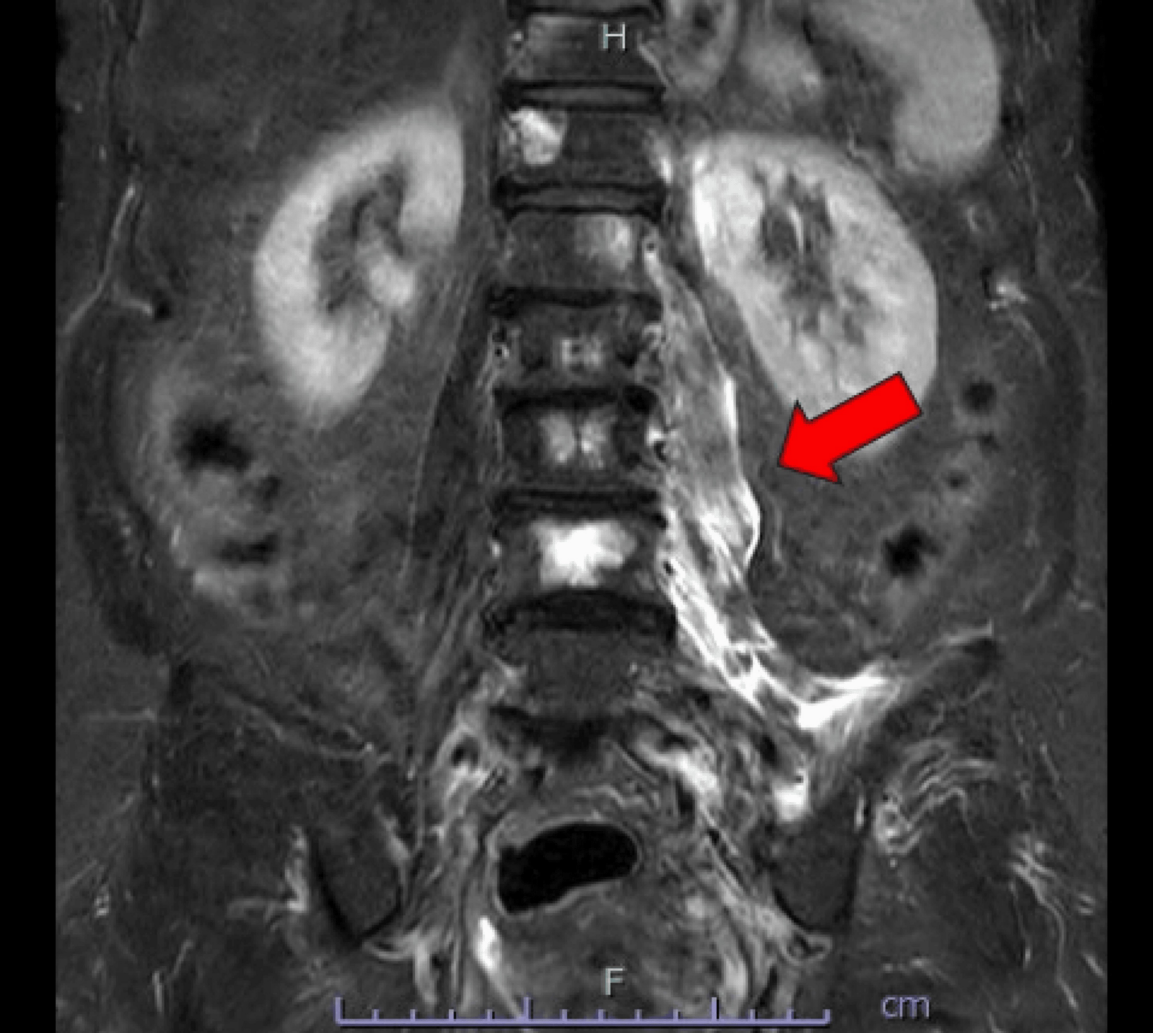
Magnetic resonance imaging findings. High signal intensity in the left psoas major and iliac muscles on short tau inversion recovery (arrow); no evidence of neurological involvement.

An iliopsoas abscess was suspected based on the CT findings; however, blood tests showed normal inflammatory markers, with a white cell count of 7830/μL (reference: 4000-10000/μL), neutrophil count of 4761/μL (reference: 2000-7000/μL), and C-reactive protein level of 0.11 mg/dL (reference: <0.30 mg/dL), making a common iliopsoas abscess unlikely. Magnetic resonance imaging (MRI) of the lumbar spine was performed to further evaluate the psoas lesion. MRI showed high signal intensity in the left psoas major and iliac muscles on short tau inversion recovery, suggesting an iliopsoas abscess (Figure 3).

**Figure 4:**
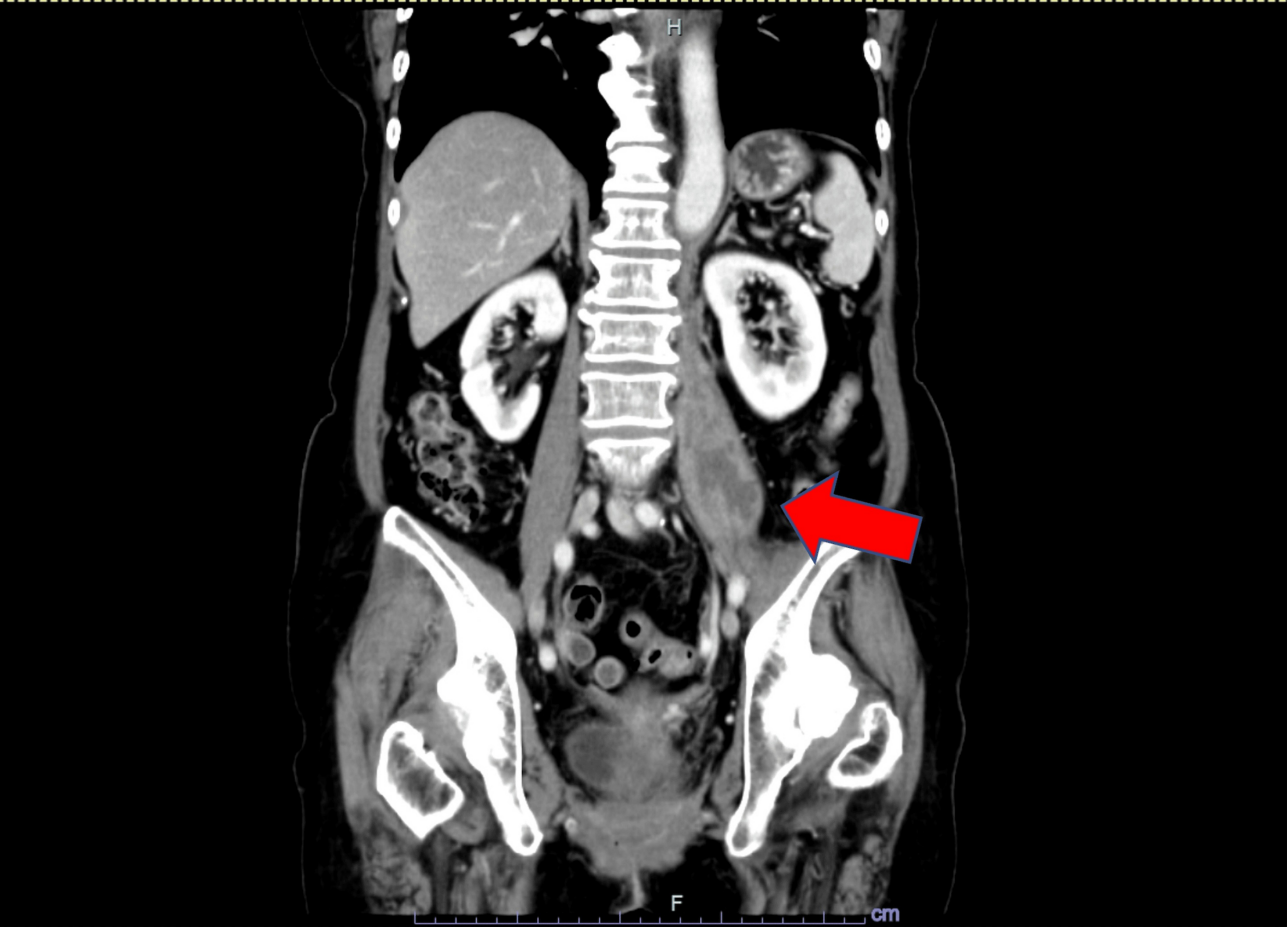
Coronal computed tomography findings. Low-density mass in the left psoas muscle with enhancing peripheral rim (arrow), measuring 67 × 32 mm.

Since the pain was mild and the radiological findings were suggestive of an abscess in the absence of systemic inflammatory response, a definitive diagnosis could not be established at that time. Therefore, the patient was managed conservatively with careful observation and close monitoring of clinical symptoms and lesion progression. Over time, the symptoms gradually worsened. Repeat CT two months later showed the mass increased 14 mm in size from the previous scan (Figure 4).

**Figure 5:**
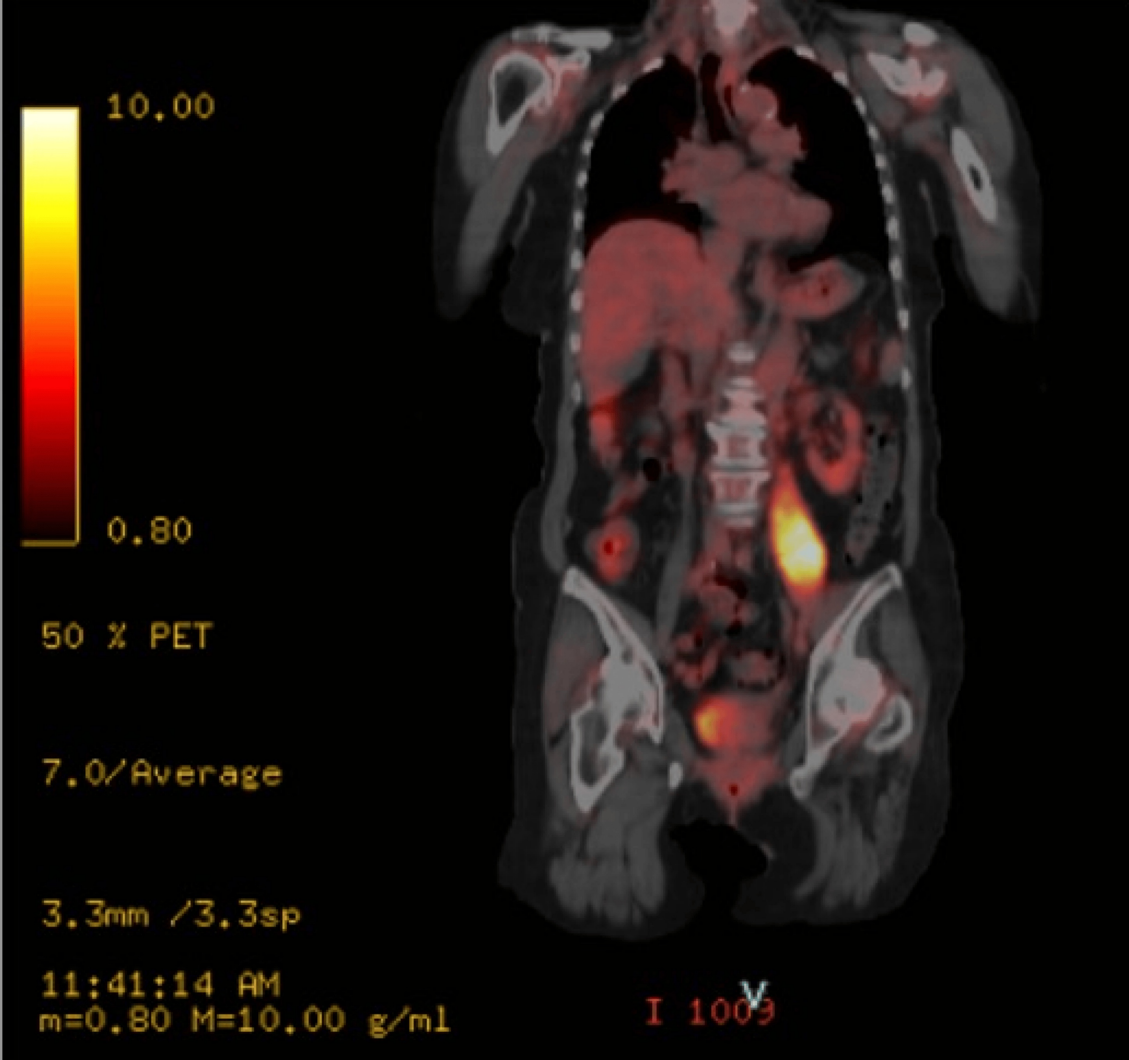
Positron emission tomography-computed tomography findings. Abnormal accumulation in the left psoas muscle (maximum standardized uptake value = 11.2), suggestive of malignancy rather than an abscess.

Positron emission tomography (PET)-CT revealed an abnormal uptake in the left psoas muscle, with a maximum standardized uptake value of 11.2 (Figure 5).

**Figure 6:**
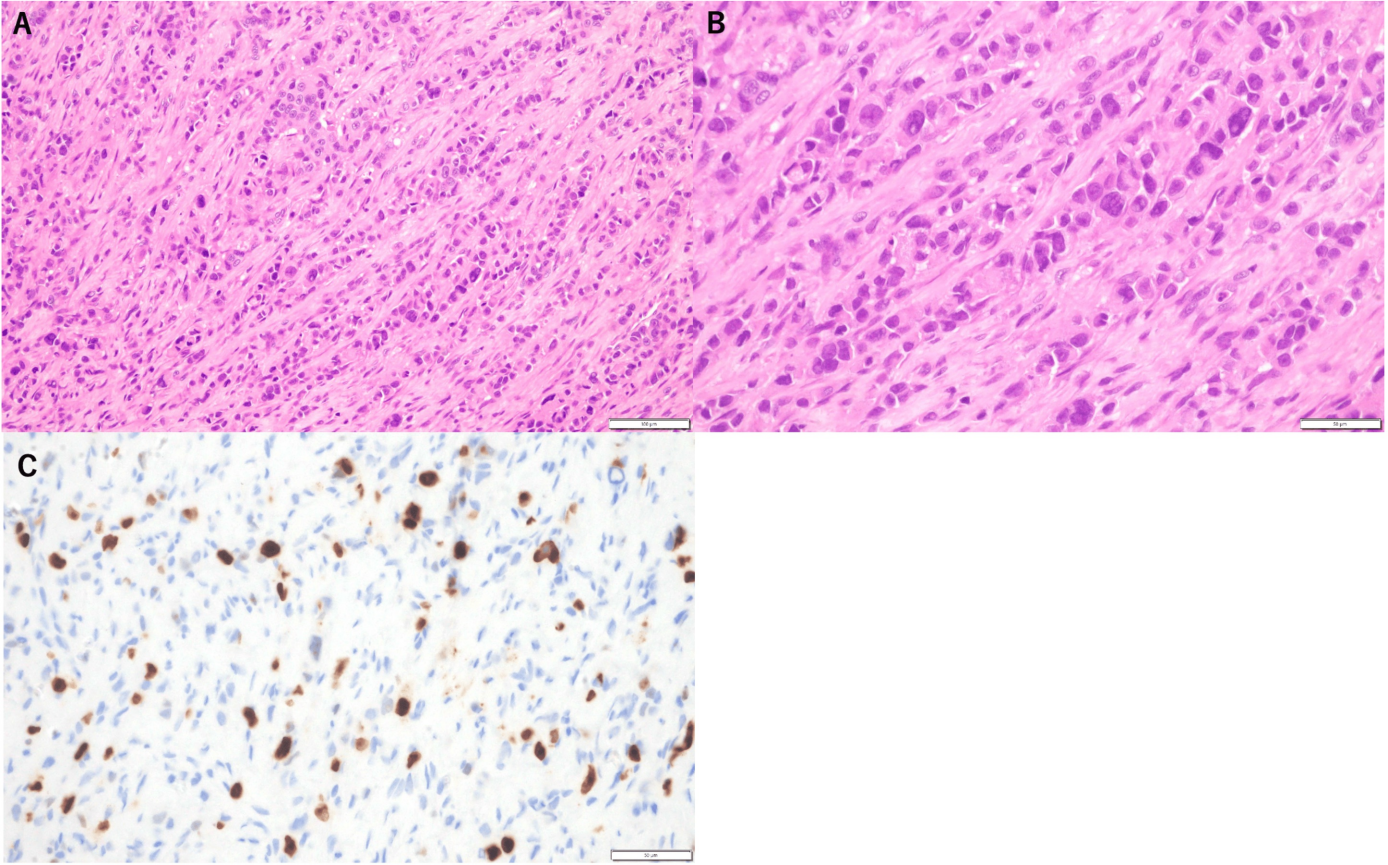
Microscopic morphology of metastasis to the psoas muscle. Diffuse scattered neoplastic cells with muscle fascicles. (A) Hematoxylin and eosin (H&E) stain (×200). (B) H&E stain (×400). (C) The MIB-1 labeling index was 8% (×400).

CT-guided biopsy revealed pathological findings consistent with invasive lobular carcinoma (ILC), with an MIB-1 labeling index of 8% (Figure 6).

IHC analysis of the biopsy specimen revealed ER-positive, PR-negative, and HER2-negative characteristics, suggestive of metastasis.

**Figure 7:**
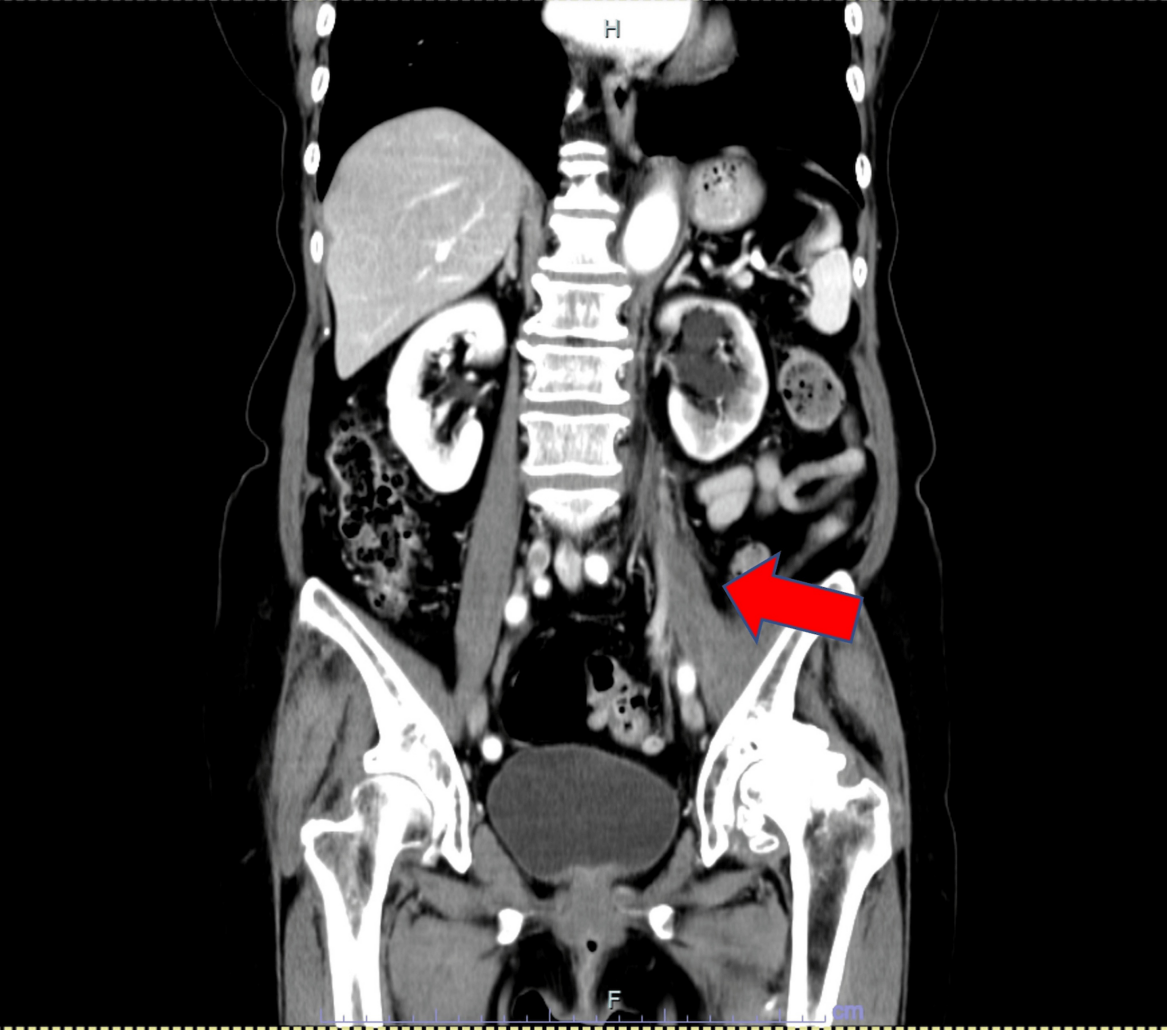
Coronal computed tomography findings. Initial treatment led to a reduction in the size of the psoas lesion to 48 × 14 mm.

The patient was diagnosed with MPS caused by a breast cancer psoas metastasis. Treatment for disease control included the CDK4/6 inhibitor palbociclib and hormone therapy with fulvestrant, resulting in a reduction in the metastatic lesions (Figure 7).

Pain was well controlled with tramadol and acetaminophen, and treatment with radiotherapy was deemed unnecessary. Based on the course of the pain, it was considered nociceptive pain. However, seven months later, a new retroperitoneal mass appeared. Chemotherapy with eribulin 1.4 mg/m^2^ was given for disease control and maintained stable disease. One year after the MPS diagnosis, the patient remains under outpatient treatment and continues to be monitored.

## Discussion

Skeletal muscle metastases from breast cancer are extremely rare, with only eight previous case reports providing detailed information (Table 1) [4-10].

**Table 1:** Clinical course in eight reported cases of muscle metastasis from breast cancer.

	Metastatic site in skeletal muscle	Histological subtype	Confirmative diagnosis procedure	Treatment	Outcome
1	Abdominal wall muscle	Invasive ductal carcinoma	PET-CT, core needle biopsy	Chemotherapy	Partial response, later progression
2	Gluteus maximus	Invasive ductal carcinoma	PET-CT, ultrasound biopsy	Endocrine therapy → chemotherapy	Partial response, under treatment
3	Abdominal wall and iliac muscle	Metaplastic carcinoma	CT scan, core needle biopsy	Radiotherapy and palliative chemotherapy	Died 10 months post metastasis
4	Forearm, psoas, quadratus lumborum	Metaplastic carcinoma	PET-CT, surgery, pathology	Surgery + palliative radiotherapy	Died 12 months post metastasis
5	Biceps brachii	Invasive ductal carcinoma	PET-CT, core needle biopsy	Endocrine therapy + palliative radiotherapy	Tumor shrinkage, symptoms improved
6	Sternocleidomastoid, trapezius	Invasive lobular carcinoma	CT scan, core needle biopsy	Endocrine therapy + palliative radiotherapy	Died 1 month post diagnosis
7	Adductor magnus muscle	Invasive lobular carcinoma	MRI, fine needle aspiration	Endocrine therapy + palliative radiotherapy	Progressive disease 1 month post diagnosis
8	Gluteal muscle	Invasive ductal carcinoma	PET-CT, core needle biopsy	Surgery + chemotherapy	No recurrence 12 months post surgery

This rarity is supported not only by the limited number of clinical reports but also by the biological resistance of muscle tissue to metastatic colonization. The inhospitable environment of skeletal muscle, characterized by mechanical tumor destruction during contraction and unfavorable pH conditions, may help explain this phenomenon [2]. However, tumor factors also play a role in the establishment of metastasis. In particular, the histological type of breast cancer has a significant influence on the type of metastasis. ILC is the second most common histological type of breast cancer following invasive ductal carcinoma and is characterized by a unique metastatic pattern compared to invasive ductal carcinoma [11]. Loss of E-cadherin expression in ILC disrupts intercellular adhesion, leading to a distinctive single-file infiltration of tumor cells and a higher propensity for diffuse dissemination [11,12].

MPS is a rare condition (affecting less than 1% of patients) characterized by severe pain associated with malignant tumors that invade or metastasize to the psoas muscle [1,3,13]. The clinical criteria for diagnosing MPS include the presence of one or more of the following features, along with CT or pathological evidence of malignant involvement of the ipsilateral psoas muscle: (1) ipsilateral nociceptive pain (commonly located in the abdomen, back, hip, or thigh); (2) ipsilateral proximal (L1-L4) neuropathic pain; and (3) psoas muscle spasms causing painful ipsilateral hip flexion [13]. In the present case, the patient met these criteria, leading to a diagnosis of MPS. To the best of our knowledge, this is the first reported case of MPS caused by breast cancer that has been pathologically confirmed. This case not only fulfills the clinical definition of MPS but also reflects the metastatic behavior characteristic of ILC.

Early recognition of MPS is essential for optimizing patient care. CT is useful for the initial detection and morphological assessment of lesions, and contrast-enhanced MRI has been reported to show extensive peritumoral enhancement and central necrosis, which are suggestive of metastatic disease [14]. However, psoas muscle metastasis can closely mimic abscesses or hematomas on imaging, making accurate differentiation difficult. In the present case, the differential diagnoses for the psoas lesion included pyogenic abscess, cold abscess (e.g., tuberculous), hematoma, and metastatic disease. Pyogenic abscess was ruled out due to the absence of systemic signs of infection and normal inflammatory markers. Although a cold abscess was considered, the lack of relevant clinical history and progressive enlargement of the lesion raised a strong suspicion for malignancy.

Histopathological examination remains the gold standard for confirming the diagnosis of psoas muscle lesions. In this case, PET-CT was also found to be highly useful in differentiating MPS from other abnormalities involving the psoas muscle [15]. While chemotherapy has shown efficacy in some reported cases [3], the overall prognosis of MPS remains poor, and palliative care often becomes the mainstay of management. Multimodal approaches to pain control are recommended [13].

## Conclusions

Psoas muscle metastasis from breast cancer is rare and may present a significant diagnostic challenge. Clinicians should consider the possibility of MPS in patients with recurrent breast cancer who develop hip or back pain. Early diagnostic measures, including biopsy and advanced imaging techniques such as PET-CT, may contribute to a more accurate diagnosis. Timely recognition of MPS in patients with advanced malignancy may lead to earlier diagnosis, better symptom control, and improved quality of life.
